# 滤膜冷凝收集-离子色谱法测定固液气溶胶中5种水溶性阴离子

**DOI:** 10.3724/SP.J.1123.2021.01007

**Published:** 2022-01-08

**Authors:** Zuosi YU, Yu LIU, Yan ZHU

**Affiliations:** 1.浙江大学化学系, 浙江 杭州 310028; 1. Department of Chemistry, Zhejiang University, Hangzhou 310028, China; 2.浙江大学浙江省微量有毒化学物健康风险评估技术研究重点实验室, 浙江 杭州 310028; 2. Key Laboratory of Health Risk Appraisal for Trace Toxic Chemicals of Zhejiang Province, Zhejiang University, Hangzhou 310028, China; 3.浙江大学生命科学院, 浙江 杭州 310058; 3. College of Life Science, Zhejiang University, Hangzhou 310058, China

**Keywords:** 离子色谱, 水溶性离子, 气溶胶, 冷凝, ion chromatography (IC), soluble ions, aerosols, condensation

## Abstract

水溶性离子是固、液气溶胶的重要组成部分,对于气溶胶的理化性质和空气质量具有重大影响,研究水溶性离子的含量对于大气环境的污染与防治具有深远意义。该研究建立了一种滤膜冷凝收集-离子色谱技术采集固体气溶胶和液体气溶胶并测定其中的5种水溶性阴离子(Cl^-^、F^-^、NO_3_^-^、NO_2_^-^、SO_4_^2-^)含量的方法。首先,采用固体颗粒过滤器和冷凝收集法分别收集固体气溶胶和液体气溶胶,固体气溶胶以固体颗粒物的形式被收集在固体颗粒过滤器内,液体气溶胶以冷凝液的形式在冷阱中被收集。其次,以离子色谱法对固、液体气溶胶中的水溶性阴离子含量进行检测。在以Dionex IonPac AS11-HC-4 μm作为分析柱,流速为1 mL/min,柱温为30 ℃,淋洗液氢氧化钾(KOH)浓度在0~40 min内由1 mol/L线性增至25 mol/L,进样量100 μL的条件下,各离子在40 min内有效分离,5种阴离子在0.1~10 mg/L范围内线性关系良好(相关系数为0.9992~0.9997),检出限低(0.02~0.04 mg/L)。对样品采集条件(采样时间、采样温度和采样流量)进行了优化,结果表明,在采样时间2 h、采样温度-13 ℃、采样流量1.0 L/min的条件下,可获得较为满意的结果。在优化的条件下分别对实际样品的两类溶胶中的5种阴离子含量进行了检测,测得实际样品的液体气溶胶中5种阴离子含量分别为5.7402 μg/m^3^ (F^-^)、1.1599 μg/m^3^ (Cl^-^)、3.3233 μg/m^3^ (NO_2_^-^)、2.4861 μg/m^3^ (NO_3_^-^)和0.9745 μg/m^3^ (SO_4_^2-^),固体气溶胶中5种阴离子含量分别为14.1037 μg/m^3^ (F^-^)、5.0398 μg/m^3^ (Cl^-^)、9.3052 μg/m^3^ (NO_2_^-^)、8.4528 μg/m^3^ (NO_3_^-^)和5.6314 μg/m^3^ (SO_4_^2-^)。该方法可应用于实际的大气检测中,也为其他离子的采集和分析条件的摸索提供了方法。

## Abstract (en)

Water-soluble ions are important components of solid and liquid aerosols. The ions have a significant impact on the physical and chemical properties of the aerosols and air quality. Thus, determining the concentrations of water-soluble ions in solid and liquid aerosols has far-reaching significance for mitigating pollution and protecting the atmospheric environment. In this study, a technology combining membrane filtration, condensation, and ion chromatography has been established to collect solid aerosols and liquid aerosols and to measure the amount of five water-soluble anions (Cl^-^, F^-^, NO_3_^-^, NO_2_^-^, and SO_4_^2-^) in these aerosols. First, a solid particle filter was used to collect the solid aerosol, and the condensation collection method was used to collect the liquid aerosol. The solid aerosol was collected in the solid particle filter in the form of solid particles, and the liquid aerosol was collected in the cold trap in the form of a condensate. Next, the amount of water-soluble anions in the solid aerosols and liquid aerosols was detected by ion chromatography using Dionex IonPac AS11-HC-4 μm as the analytical column. The chromatographic separation conditions were as follows: flow rate, 1 mL/min; column temperature, 30 ℃; potassium hydroxide (KOH) concentration of the eluent, 1 to 25 mol/L (linear increase) within 0-40 min; and injection volume, 100 μL. The ions were separated effectively within 40 min. The five anions showed good linear relationships in the range of 0.1-10 mg/L (correlation coefficients ranged from 0.9992 to 0.9997) and the detection limits were 0.02-0.04 mg/L. Finally, the sample collection conditions (sampling time, sampling temperature, and sampling flow rate) for the five water-soluble anions in the solid aerosols and liquid aerosols were optimized, and 2 h, -13 ℃, 1.0 L/min were chosen.Under the optimized conditions, the amounts of the five anions in the solid and liquid aerosols of the actual samples were detected. The mass concentrations of F^-^, Cl^-^, NO_2_^-^, NO_3_^-^, and SO_4_^2-^ ions in the liquid aerosol of the actual sample were 5.7402 μg/m^3^, 1.1599 μg/m^3^, 3.3233 μg/m^3^, 2.4861 μg/m^3^, and 0.9745 μg/m^3^, respectively. The mass concentrations of F^-^, Cl^-^, NO_2_^-^, NO_3_^-^, and SO_4_^2-^ ions in the solid aerosol of the actual sample were 14.1037 μg/m^3^, 5.0398 μg/m^3^, 9.3052 μg/m^3^, 8.4528 μg/m^3^, and 5.6314 μg/m^3^, respectively. The method can be applied to the detection of actual atmospheric ions detection. In addition, the collection and analysis methods are suitable for exploring the collection and analysis conditions of other ions.

大气溶胶主要由悬浮在空气中的固体颗粒和液滴组成,可以分为固体气溶胶和液体气溶胶,两类气溶胶的组成、粒径大小等在空间和时间的分布有着很大不同^[1,2]^。水溶性离子是固、液气溶胶的重要组成部分,这类物质对于气溶胶的分布、含量、酸碱度及化学组成等有着很大的影响^[3,4,5]^,进而影响着空气质量和空气能见度,同时对于环境和人体的健康有着重要影响^[6,7,8]^。因此,研究大气溶胶中的水溶性离子对于研究大气溶胶的物理特性与化学组成具有重要意义,同时对于环境保护和污染防治有深远的指导意义。

目前对于气溶胶中水溶性离子的相关研究较多,但主要涉及大气溶胶中水溶性离子方面的研究,其中滤膜收集法等方法广泛应用于大气溶胶的收集^[9,10,11]^。方言等^[12]^使用石英滤膜收集样品,离子色谱法来分析气溶胶中的水溶性离子的质量浓度,探讨了东海背景站水溶性离子的化学特征等。侯忠新等^[13]^用KC-1000采样器(微孔滤膜)进行采样,研究了青岛地区的气溶胶质量浓度和水溶性离子的分布特征。张程等^[14]^利用Andersen型9级撞击式采样器(石英微孔膜)对气溶胶采样,利用离子色谱分析了气溶胶中的水溶性离子的变化、分布和来源等特征。另外,对固体类气溶胶中水溶性离子的研究也比较多,这里主要是对于PM_2.5_和PM_10_作为研究对象。雷天阳等^[15]^使用TH-16A型采样器和石英滤膜对PM_2.5_进行采集,利用离子色谱分析其中水溶性离子的化学特征,并对采集地点的污染特征进行了分析。刀谞等^[16]^使用TH-16A采样器对PM_2.5_和PM_10_进行采集,剖析了京津冀地区的颗粒物浓度和9种水溶性阴离子的浓度高低规律。

固体气溶胶中水溶性离子的种类和含量可反映大气污染物的种类和来源,可在一定程度上反映采集地的污染物水平^[17]^。液体气溶胶中水溶性离子与相对湿度和能见度呈现相关性,其气候环境效应对于雾天、轻雾天等天气的形成有重要的影响^[18]^。传统的大气采集仪器仅可以收集空气中的固、液气溶胶的总量或者单独收集某种尺寸的固体颗粒物(固体气溶胶),没有单独实现固、液气溶胶的同步分离收集,要测定液体气溶胶的水溶性离子含量,需先测定气溶胶中离子的总含量,再测定固体颗粒物中离子含量,然后才能间接得到实验结果。搭建能同步收集固体气溶胶和液体气溶胶的采集装置能简化采集步骤,优化实验结果,并能达到直接测定液体气溶胶中水溶性离子含量的目的。

本研究利用固体颗粒过滤器和冷凝的方法同时分别采集固、液气溶胶,采用离子色谱,选择Dionex IonPac AS11-HC-4 μm色谱柱对5种水溶性阴离子进行分析,并对采集条件进行优化。

## 1 实验部分

### 1.1 仪器与试剂

DIONEX ICS 5000^+^DP、ASRS-3000 4 mm型阴离子抑制器、DIONEX ICS-3000 EG DIONEX ICS-3000 DC和Dionex IonPac AS11-HC-4 μm色谱分析柱购自美国Thermo Fisher Scientific公司;便携式ZC-Q型大气采样器购自浙江恒达仪器股份有限公司;半导体制冷装置、保温铝材和样品采样管、微孔滤膜(直径50 mm,孔径1 μm)和固体颗粒过滤器(内径50 mm)均为实验室定制。

氢氧化钾、氯化钠、亚硝酸钾、硝酸钾、无水硫酸镁和氟化铵均为分析纯,购于阿拉丁公司。

### 1.2 样品采集

采集地点:浙江大学西溪校区西七操场。在大气采样器的抽气口前分别连接固体颗粒过滤器、半导体冷凝装置和气体缓冲装置。通过大气采样器抽气,使气体从半导体冷凝装置的冷凝收集管的入气口先进入固体颗粒过滤器,过滤掉大气气溶胶中的固体气溶胶,然后进入半导体冷凝收集装置的冷阱中,液体气溶胶会在冷阱中的冷凝收集管中液化,难液化的气体最后经过气体缓冲装置进入采样器的泵中。装置连接示意图见图1。该收集装置可以改变温度和采样流量对大气进行采样,收集后的样品主要分为两部分,一部分是固体颗粒过滤器中滤膜上收集到的固体气溶胶,另一部分是在冷凝收集管中的液体气溶胶。将固体颗粒过滤器中的滤膜取出,置于30 mL的超纯水中超声浸泡30 min以上,收集浸泡后的溶液保存。同时从冷阱中取出冷凝收集管,保存收集管中的冷凝液。

**图 1:**
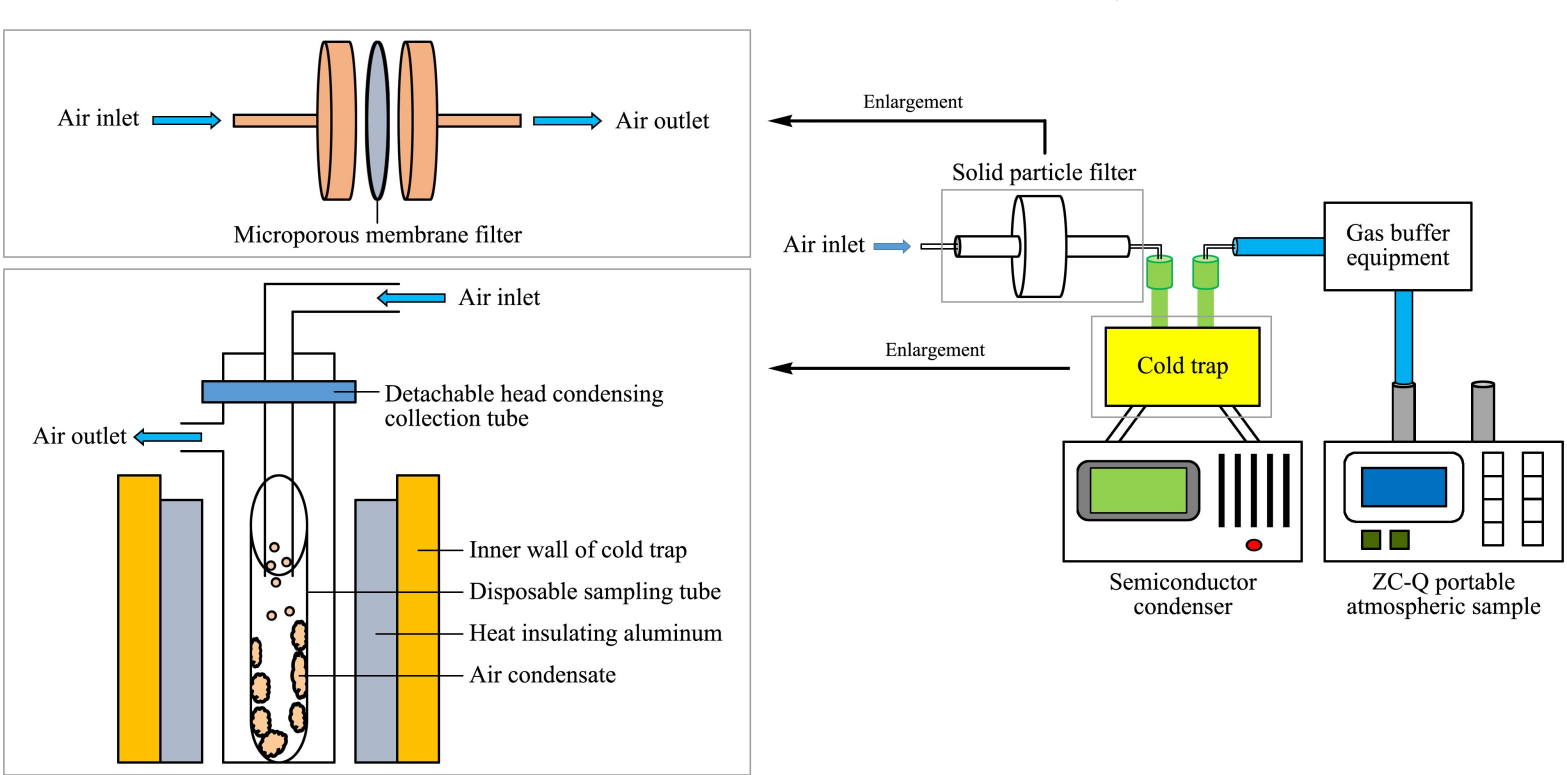
固、液气溶胶采集装置示意图

### 1.3 色谱条件

色谱柱:Dionex IonPac AS11-HC-4 μm分析柱;流速:1 mL/min;柱温:30 ℃;淋洗液:KOH梯度淋洗,0~40 min内浓度由1 mol/L线性增加到25 mol/L;进样量:100 μL。

### 1.4 标准溶液曲线绘制

取氢氧化钾、氯化钠、亚硝酸钾、硝酸钾、无水硫酸镁和氟化铵固体,用蒸馏水配制得到Cl^-^、F^-^、 ${\mathrm{NO}}_{3}^{-}$ 、 ${\mathrm{NO}}_{2}^{-}$ 、 ${\mathrm{SO}}_{4}^{2-}$ 单个离子的标准溶液(质量浓度均为100 mg/L),然后再分别配制质量浓度为0.1、1、2、5和10 mg/L的混合标准溶液。在1.3节色谱条件下测定各峰面积,以质量浓度和峰面积为函数关系绘制标准曲线。

### 1.5 样品分析与含量计算

将浸泡液、冷凝液和去离子水空白样品通过微孔滤膜过滤处理后进行离子色谱分析,样品色谱图扣除空白样的色谱图后得到各离子的峰面积,结合标准曲线计算样品中的各离子含量。然后根据公式(1)、(2)、(3)、(4)分别计算冷凝液中的水溶性离子质量*C*_1_、每立方米大气中液体气溶胶中的水溶性离子含量*C*_2_、浸泡液中的水溶性离子质量*C*_3_、每立方米大气中固体气溶胶中的水溶性离子含量*C*_4_。通过*C*_1_、*C*_3_的增量变化研究采样过程中收集效率的变化与影响,*C*_2_、*C*_4_在本实验中作为采集条件优化判断的指标,在实际的大气监测中是评价污染物浓度的重要指标。


(1) *C*_1_=*C*_0_*V*_1_

(2) $C_{2}=\frac{C_{0} V_{1}}{V_{0}}$

(3) *C*_3_=*CV*_2_

(4) $C_{4}=\frac{C V_{2}}{V_{0}}$

其中,*C*_0_为收集的冷凝液中水溶性阴离子的质量浓度,mg/L; *V*_1_为冷凝物的体积,mL; *C*为收集的浸泡液中水溶性阴离子的质量浓度,mg/L; *V*_2_为浸泡液的体积,mL; *V*_0_为标准状态下的采样体积,m^3^。

## 2 结果与讨论

### 2.1 标准曲线与检出限

将一系列混合标准溶液进样,扣除空白色谱峰,以质量浓度为*x*,峰面积为*y*,绘制标准曲线;以3倍信噪比确定检出限,结果见表1。

**表 1:** 标准曲线、线性范围、相关系数(*r*^2^)与检出限

Ion	Standard curve	Linear range/ (mg/L)	r^2^	LOD^*^/ (mg/L)
F^-^	y=0.9069x+0.0369	0.1-10	0.9997	0.02
Cl^-^	y=0.8739x+0.0109	0.1-10	0.9992	0.02
N	y=0.4824x-0.0350	0.1-10	0.9993	0.04
N	y=0.6127x-0.0046	0.1-10	0.9996	0.03
S	y=0.6992x-0.0226	0.1-10	0.9995	0.04

*y*: peak area; *x*: mass concentration, mg/L; * *S/N*=3.

### 2.2 采样时间的优化

设置采样时间分别为1、2、3、4和5 h,采样温度为-5 ℃,采样流量为1 L/min的条件进行采集并检测,各离子含量与时间的关系图见图2。

**图 2:**
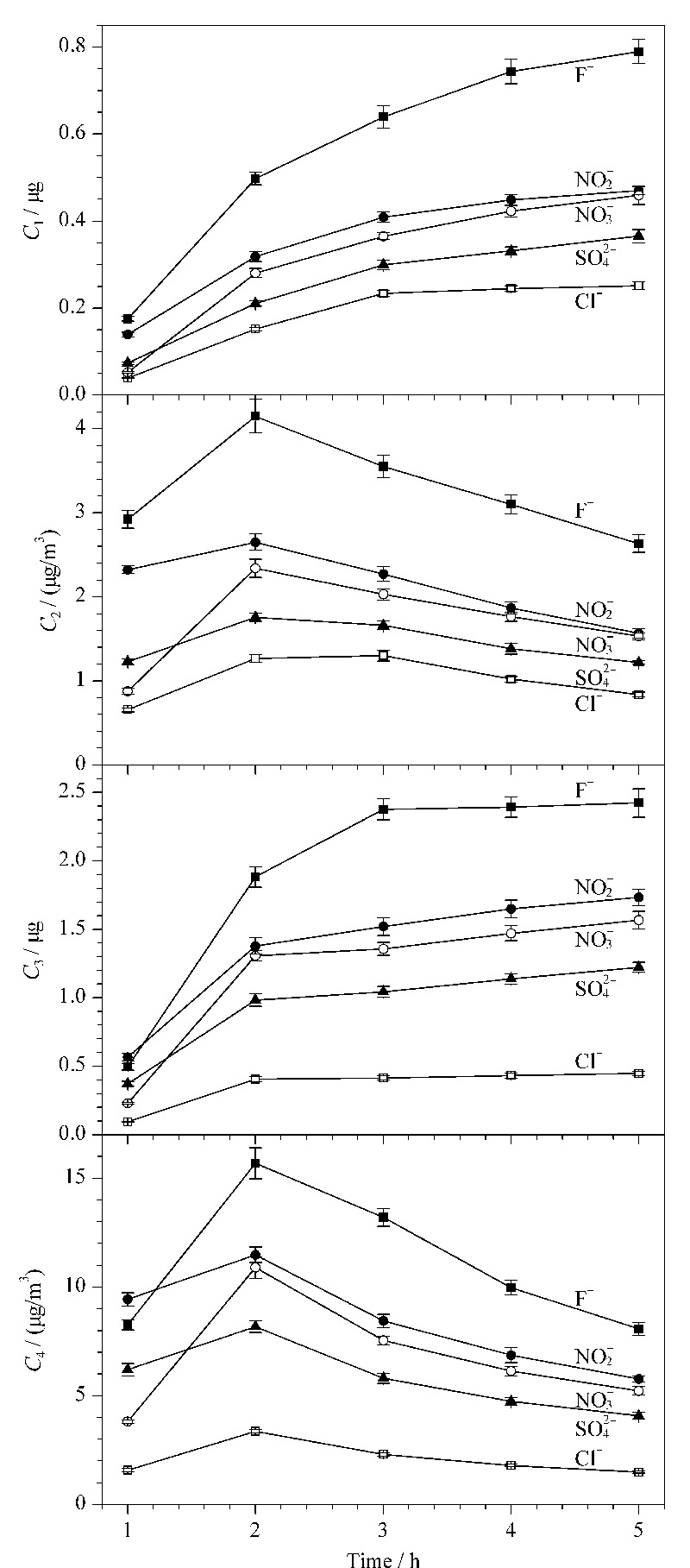
不同采样时间下的*C*_1_~*C*_4_(*n*=5)

从图2可以发现,采样时间增加,*C*_1_和*C*_3_不断增大,但由折线斜率得到增长的速率不断减小,尤其是*C*_3_在3 h后基本没有增加,这可能是固体颗粒过滤器中微孔滤膜在收集过程中表面颗粒不断增加导致吸附效率减小所造成的。5种离子的*C*_2_和*C*_4_有着相似的规律,都随收集时间的增大,其含量呈先增大后减小的趋势,其中各离子的*C*_2_和*C*_4_都在采样时间为2 h时最大。因此,2 h是5种离子的最优采样时间。

### 2.3 采样流量的优化

设置采样流量分别为0.5、1、1.5和2 L/min,采样时间2 h,采样温度为-5 ℃的条件进行采集并检测,各含量与采样流量的关系图见图3。*C*_1_随采样流量先增大后减小表明,流量过大或过小均不利于液体气溶胶的采样效率的提高,5种阴离子在流量1.5 L/min的条件下达到最大。采样流量大于1.5 L/min时增大流量,*C*_3_的含量变化不大,因此该情况下对固体气溶胶采样效率的影响不再明显。*C*_2_随采样流量先增后减,5种离子*C*_2_的最大值取得条件不同,因此5种离子的液体气溶胶的最优采样流量各不相同。*C*_4_变化趋势先增后减, ${\mathrm{SO}}_{4}^{2-}$ 、Cl^-^、 ${\mathrm{NO}}_{3}^{-}$ 、 ${\mathrm{NO}}_{2}^{-}$ 的*C*_4_在采样流量1.0 L/min时最大,而F^-^在采样流量1.5 L/min时最大。综合考虑,为了达到能够多种离子一次采样和进样分析的目的,选择采样流量1.0 L/min作为最优采样流量。

**图 3:**
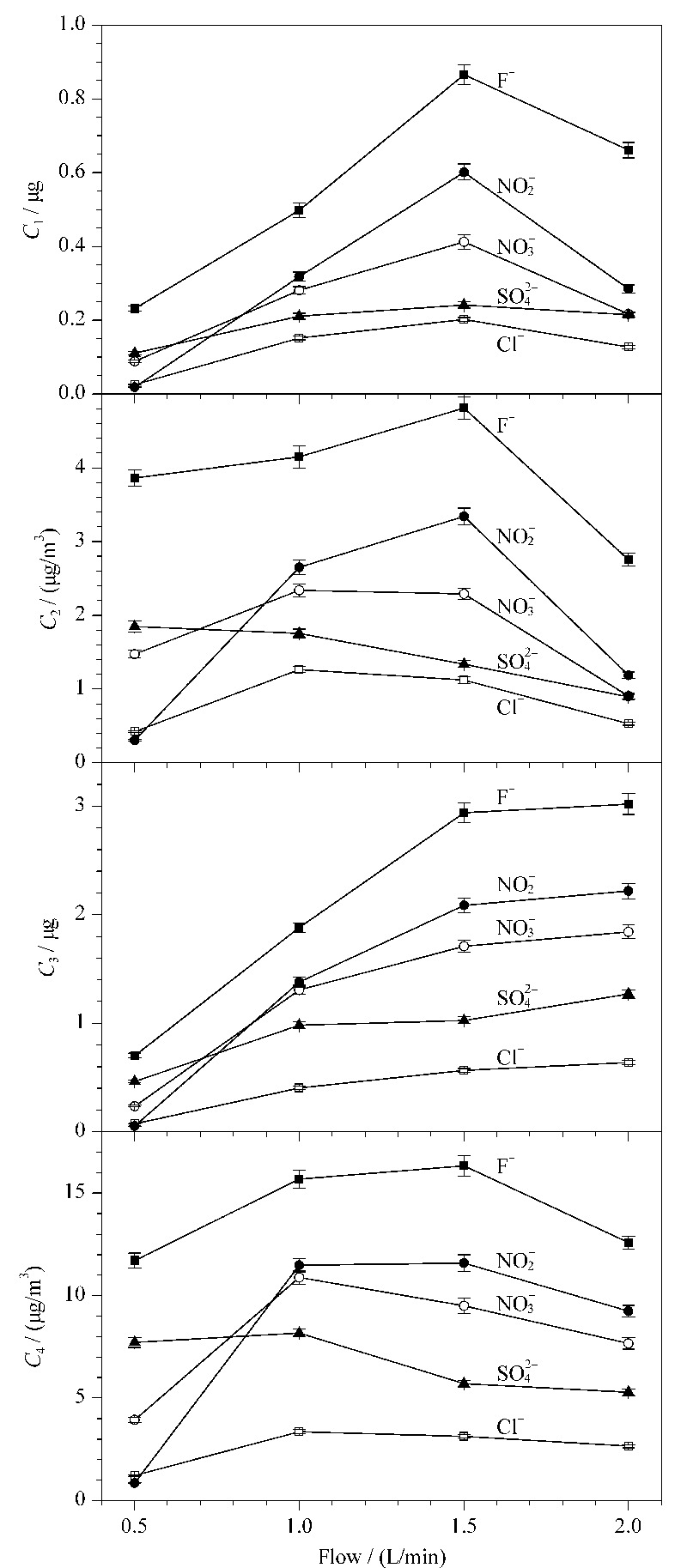
不同采集流量下的*C*_1_~*C*_4_(*n*=5)

### 2.4 温度的优化

设置采样温度分别为-1、-5、-9和-13 ℃(制冷器的极限温度是-15 ℃),采样时间2 h,采样流量为1 L/min的条件进行采集并检测,每个条件实验5次取平均值,各含量与温度的关系图见图4。

**图 4:**
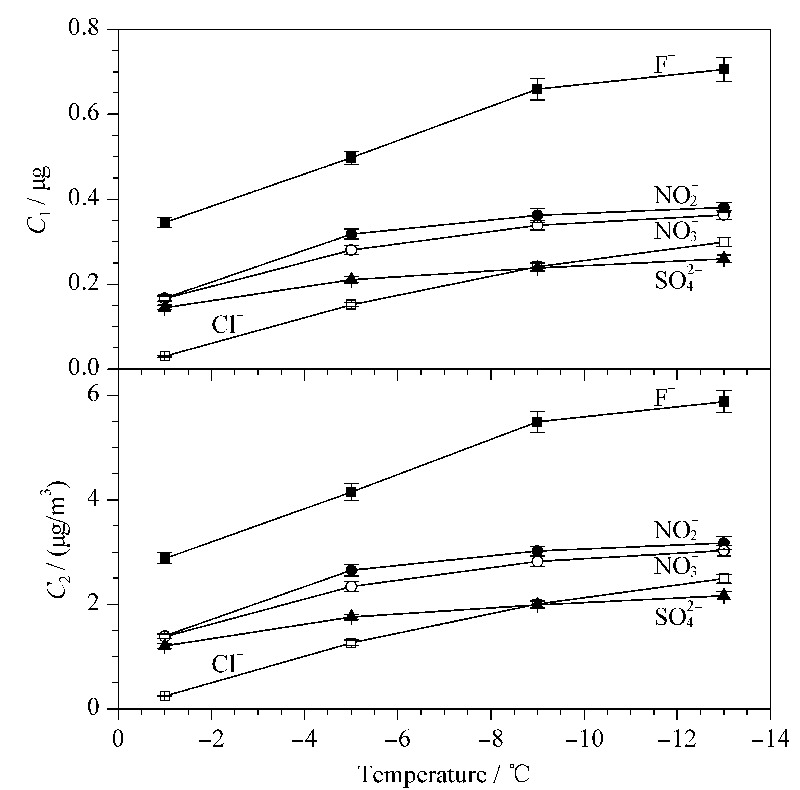
不同采集温度下的*C*_1_、*C*_2_(*n*=5)

从图4可以看到,*C*_1_和*C*_2_随采样温度下降而增大。因采样过程中固体颗粒过滤器不需要冷凝处理,所以*C*_3_和*C*_4_不受采样温度的影响。因此,采样温度-13 ℃是5种离子的最优采样温度。

### 2.5 实际样品的采集应用

在2.2~2.4节优化后的条件(采样时间2 h,温度-13 ℃,采样流量1.0 L/min)下,选择浙江大学玉泉校区第八教学楼作为采样地点进行样品采集,实验重复5次。两类气溶胶实际样品的色谱图见图5。测得的*C*_2_、 *C*_4_值及RSD见表2。样品采集得到的*C*_2_、*C*_4_即为采集地的液、固体气溶胶中5种水溶性阴离子的浓度。

**图 5:**
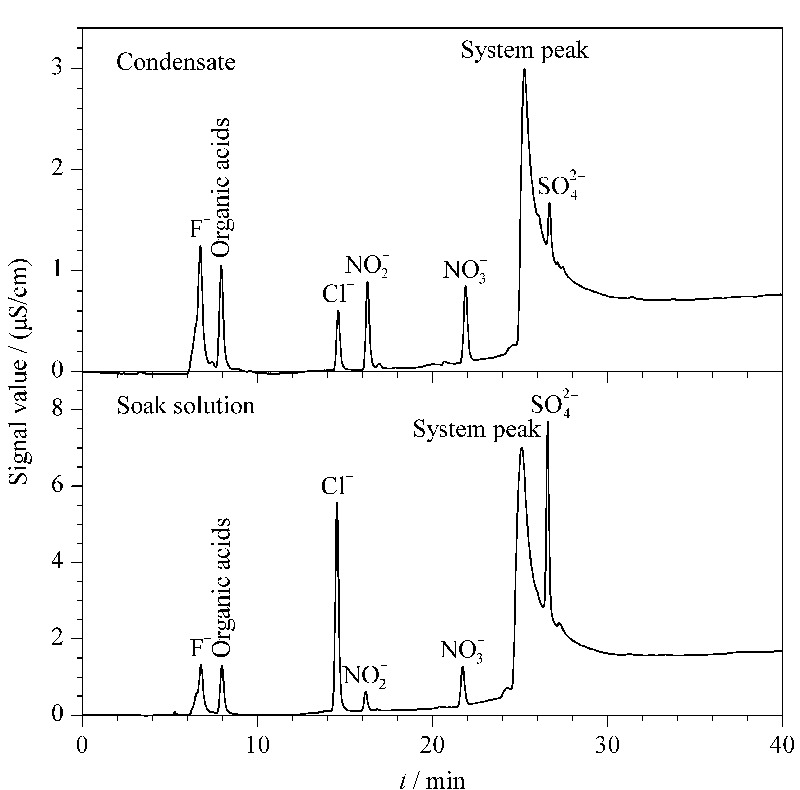
实际样品的色谱图

**表 2:** 实际样品的*C*_2_、*C*_4_及其RSD (*n*=5)

Ion	C_2_/(μg/m^3^)	C_2_ RSD/%	C_4_/(μg/m^3^)	C_4_ RSD/%
F^-^	5.7402	4.5	14.1037	3.8
Cl^-^	1.1599	4.1	5.0398	4.2
N	3.3233	3.6	9.3052	4.4
N	2.4861	3.9	8.4528	3.6
S	0.9745	3.7	5.6314	3.9

## 3 结论

建立了一种同时采集固体气溶胶和液体气溶胶的方法,利用离子色谱对两种气溶胶中的水溶性阴离子(Cl^-^、F^-^、 ${\mathrm{NO}}_{3}^{-}$ 、 ${\mathrm{NO}}_{2}^{-}$ 、 ${\mathrm{SO}}_{4}^{2-}$ )进行含量检测。优化了采样时间、采样温度和采样流量等采集条件,优化后的采集条件可作为当地气溶胶采样的收集条件,测得的两类溶胶的含量可用于评价大气环境监测过程中5种阴离子的浓度水平。该方法收集操作简便,检测手段能同时检测多种离子,是大气采样手段的一种创新。但该方法的局限在于不同的采集地点可能存在不同种类的离子,需要对采集条件重新优化。
